# Body Mass Index, Diabetes, and Risk of Tuberculosis: A Retrospective Cohort Study

**DOI:** 10.3389/fnut.2021.739766

**Published:** 2021-12-01

**Authors:** Hayoung Choi, Jung Eun Yoo, Kyungdo Han, Wonsuk Choi, Sang Youl Rhee, Hyun Lee, Dong Wook Shin

**Affiliations:** ^1^Division of Allergy, Pulmonary and Critical Care Medicine, Department of Internal Medicine, Hallym University Kangnam Sacred Heart Hospital, Hallym University College of Medicine, Seoul, South Korea; ^2^Department of Family Medicine, Healthcare System Gangnam Center, Seoul National University Hospital, Seoul, South Korea; ^3^Department of Statistics and Actuarial Science, Soongsil University, Seoul, South Korea; ^4^Department of Internal Medicine, Chonnam National University Hwasun Hospital, Chonnam National University Medical School, Hwasun, South Korea; ^5^Department of Endocrinology and Metabolism, Kyung Hee University School of Medicine, Seoul, South Korea; ^6^Division of Pulmonary Medicine and Allergy, Department of Internal Medicine, Hanyang University College of Medicine, Seoul, South Korea; ^7^Department of Family Medicine, Samsung Medical Center, Sungkyunkwan University School of Medicine, Seoul, South Korea; ^8^Department of Digital Health, Samsung Advanced Institute for Health Sciences & Technology, Sungkyunkwan University, Seoul, South Korea

**Keywords:** diabetes mellitus, tuberculosis, body mass index, epidemiology, nutritional status

## Abstract

Although both diabetes mellitus (DM) and underweight are associated with increased risk of tuberculosis (TB), there are limited data evaluating TB risk while considering two factors simultaneously—body mass index (BMI) and DM. A retrospective cohort study was performed with 10,087,903 participants of the Korean National Health Screening Program in 2009. The cohort was followed up to the date of TB incidence, death, or until December 31, 2018. We compared the incidence and risk of TB according to BMI category and DM. During the 7.3-year follow-up duration, the incidence of TB was 0.92 per 1,000 person-years in the normal weight without DM, 2.26 in the normal weight with DM, 1.80 in the underweight without DM, and 5.35 in the underweight with DM. Compared to the normal weight without DM, the normal weight with DM, the underweight without DM, and the underweight with DM showed a 1.51-fold (95% CI, 1.46–1.57), a 2.21-fold (95% CI, 2.14–2.28), and a 3.24-fold (95% CI, 2.95–3.56) increased risk of TB, respectively. However, compared to the normal weight without DM, the severely obese without DM and those with DM showed a 0.37 (95% CI, 0.36–0.38) and a 0.42 (95% CI, 0.36–0.48)-fold decreased risk of TB, respectively. There was no significant joint effect of BMI and DM on the risk of incident TB in the overall population; a synergistic effect of underweight and DM was evident in participants <65 years of age, current smokers, and heavy drinkers. In conclusion, being underweight or DM individually increases the risk of incident TB. Based on our study results, a focused screening of incident TB in patients with DM may be beneficial.

## Introduction

Worldwide, tuberculosis (TB) is a communicable disease that is a major cause of ill health and the leading cause of death from a single infectious agent (1, 2). TB is curable and preventable—the number of people developing infections and disease (and thus the number of deaths) can also be reduced through multispectral action to address TB determinants (1).

Whereas, obesity is known to increase the risk of various types of infections (3), being underweight predisposes patients to the development of TB (4). This suggests that the impact of body weight on TB risk is opposite to the impact of body weight on other infections. A previous systematic review of six cohort studies also showed an inverse relationship between body mass index (BMI) and TB development, and the incidence of TB was lower in the overweight and obese populations than in those with normal weight (4).

The inverse association between BMI and TB interestingly presents a paradox with regard to diabetes mellitus (DM) (5). Obesity is a major determinant of DM (6, 7), and DM is a well-known risk factor for TB (3, 8, 9). A systematic review comparing 13 studies examining the association between DM and TB found that diabetic patients had about a three-fold increased risk of developing TB when compared to those without DM (8). Accordingly, we may conclude that the obese population should have an increased risk of TB because they are also more likely to have DM; however, this is not consistent with available epidemiological data (5, 10).

Better understanding of the complex interplay between BMI and DM on the risk of TB has critical implications for global health. However, there is a lack of research focusing on the interaction between these two risk factors—DM and BMI—for TB development, although DM and BMI may endocrinologically interact with each other. We found two Asian studies that examined the joint effect of DM and BMI on incident TB (5, 10). However, they did not use the Asian-Pacific classifications of BMI, which limits interpretation of the studies, because the BMI cut-off points for defining overweight and obesity should be lower for Asians (11). Additionally, one study defined DM by self-reporting, which might result in under-reporting or misclassification (10). They also had limitations due to small study populations and numbers of cases [e.g., 491 incident TB cases among 167,392 participants (5)] and did not fully adjust for potential confounding factors such as socioeconomic status, including income status (5). Considering there is still uncertainty in the complex interaction between BMI and DM regarding the risk of TB, a confirmatory study on the issue is warranted.

Therefore, in the present study, using a nationwide population-based data in Korea, we aimed to investigate the contributions of BMI and DM to future TB risk while taking into consideration the potential interactions between BMI and DM.

## Materials and Methods

### Study Population and Design

We conducted a retrospective cohort study using the Korean National Health Insurance Service (NHIS) database. South Korea has a single-payer universal health system, and the NHIS maintains claims data on all reimbursed inpatient and outpatient visits, procedures, and prescriptions (12). Additionally, the NHIS database includes data from annual or biennial health screening exams provided free of charge by the Ministry of Health and Welfare. Approximately 72% of all eligible persons underwent screening from 2011 to 2014 (13).

**Figure 1:**
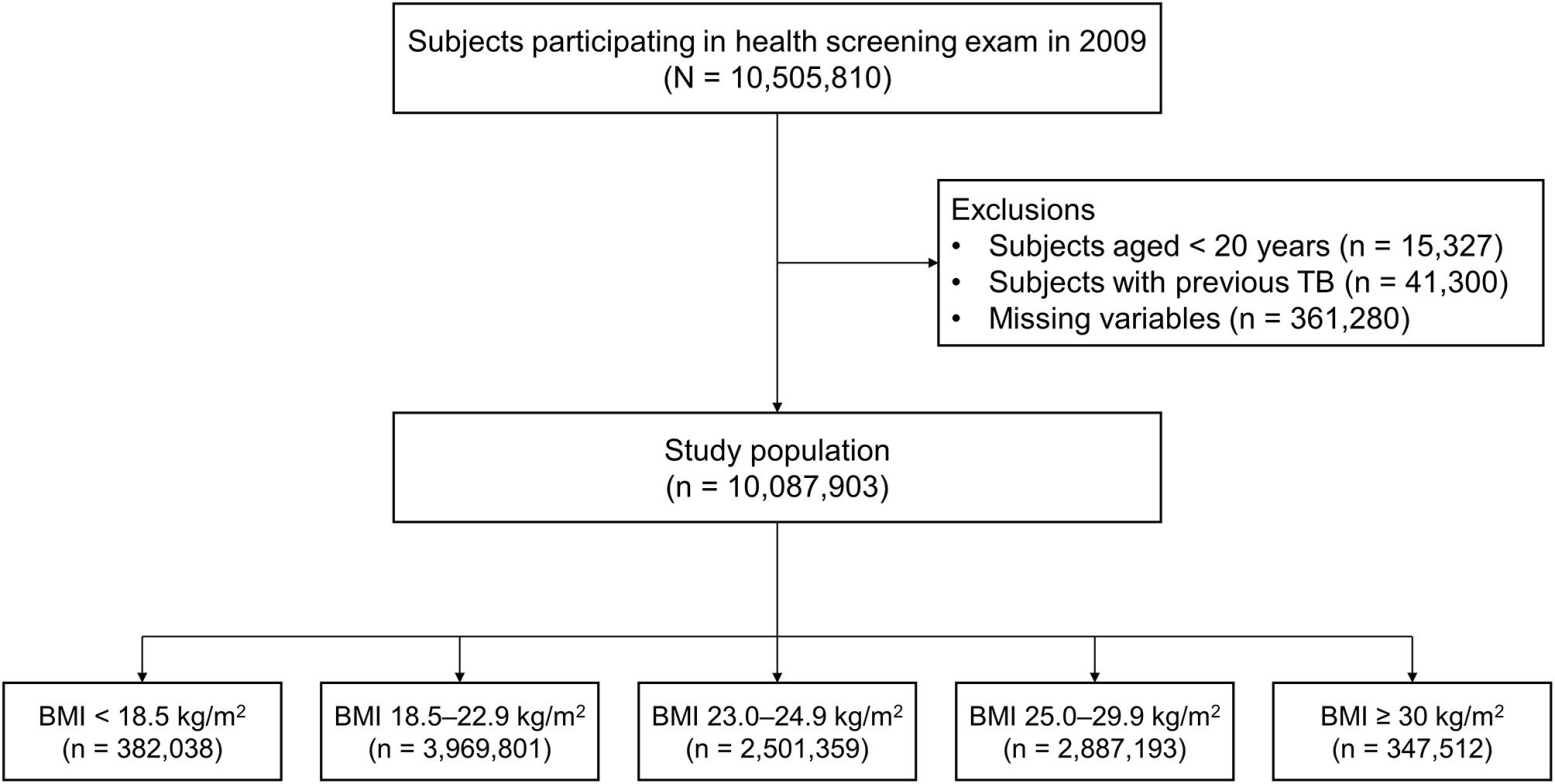
Flow chart of the study population. TB, tuberculosis; BMI, body mass index.

This study initially included 10,505,810 subjects who participated in the health screening exam between January 1, 2009, and December 31, 2009. We excluded participants who were aged less than 20 years (*n* = 15,327), those previously diagnosed with TB before the enrollment (*n* = 41,300), and those who had missing data (*n* = 361,280). Finally, a total of 10,087,903 participants were included in this study (Figure 1). The cohort was followed from baseline to the date of incident TB, death, or until the end of the study period (December 31, 2018), whichever came first.

The institutional review board of Hallym University Kangnam Sacred Heart Hospital approved the study and waived the requirement for informed consent because the NHIS database was constructed after anonymization (application no. 2020-08-015).

### Exposure: BMI and DM

Height and weight were measured in medical institutes during the health examinations. BMI was calculated as body weight (kg) divided by height (m) squared and was classified into five categories—underweight (<18.5 kg/m^2^), normal weight (18.5–22.9 kg/m^2^), overweight (23.0–24.9 kg/m^2^), obese (25.0–29.9 kg/m^2^), and severely obese (≥30 kg/m^2^)—according to the Asia-Pacific BMI criteria established by the Western Pacific Region of the World Health Organization (11).

The presence of DM was defined using International Classification of Diseases, 10th Revision (ICD-10) diagnosis codes E11–E14 plus a prescription history of hypoglycemic agents (14). Regarding DM control status, the well-controlled status was defined as fasting blood glucose <130 mg/dl and poorly controlled status was defined as fasting blood glucose ≥ 130 mg/dl (15).

### Outcome: Tuberculosis

The primary outcome of this study was incident TB, which was defined using the rare intractable disease (RID) registration codes for TB (V206, V246, and V000). All physicians should report to the public health center when they diagnose TB or suspected TB cases. The notification data include the patients' personal information, examination results, treatments, and treatment outcomes. These patients are also registered in the RID program (16). Hence, these clinical settings allowed us to identify every incident TB in the whole Korean population.

### Covariates

Information on participants' lifestyles was obtained from the health screening program self-questionnaire. Smoking status was classified into never, former, and current smoker categories. Those who consumed over 30 g of alcohol per day were defined as heavy drinkers. Regular physical activity was defined as when individuals performed strenuous exercise for at least 20 min three times per week or moderate exercise for at least 30 min five times per week (14).

Regarding comorbidities, hypertension, and dyslipidemia were defined using the ICD-10 diagnosis codes I10–I15 and E78, respectively (17).

### Statistical Analysis

Analysis of variance and a chi-squared test for continuous and categorical variables, respectively, were performed to examine the differences among BMI categories. The incidence rate of TB was calculated by dividing the number of incident cases by the total follow-up duration (1,000 person-years). Cox proportional hazards regression analyses were used to evaluate the association of BMI with the incidence of TB, without considering the time-varying nature of BMI. The multivariable model was fully adjusted for age, sex, smoking status, alcohol consumption (heavy drinker or not), regular physical activity, income (lowest 20% or not), hypertension, and dyslipidemia. The analyses were stratified by the presence of DM, along with sex and age group (<65 or ≥65 years). The joint effects of DM and BMI on TB development were assessed, and the effects were also assessed in subgroups that were stratified according to age, sex, smoking status, and alcohol consumption (18). Statistical analyses were performed using SAS version 9.4 (SAS Institute Inc., Cary, NC, USA). All tests were two-sided, and *P*-values < 0.05 were considered statistically significant.

## Results

### Baseline Characteristics

Table 1 depicts the baseline characteristics of the study population according to BMI category. The distribution of the total study cohort was as follows: 3.8% were underweight (<18.5 kg/m^2^), 39.4% were normal weight (18.5–22.9 kg/m^2^), 24.8% were overweight (23.0–24.9 kg/m^2^), 28.6% were obese (25.0–29.9 kg/m^2^), and 3.4% were severely obese (≥30 kg/m^2^). Underweight participants were more likely to be younger (mean ± standard deviation, 40.8 ± 16.8 years) and never smokers (70.1%) compared with those in other BMI categories. Additionally, underweight participants were less likely to be males (33.6%), heavy drinkers (4.5%), perform regular physical activity (9.9%), and have comorbidities including DM (3.5%), hypertension (10.4%), and dyslipidemia (5.5%) compared with those in other BMI categories.

**Table 1:** Baseline characteristics of study population.

	**Body mass index**					
	** <18.5 kg/m^**2**^**	**18.5–22.9 kg/m^**2**^**	**23.0–24.9 kg/m^**2**^**	**25.0–29.9 kg/m^**2**^**	**≥30 kg/m^**2**^**	***P*-value**
*N* (%)	382,038 (3.8)	3,969,801 (39.4)	2,501,359 (24.8)	2,887,193 (28.6)	347,512 (3.4)	
Age, years	40.8 ± 16.8	45.4 ± 14.4	48.8 ± 13.4	49.1 ± 13.3	46.3 ± 13.9	<0.001
Male	128,436 (33.6)	1,895,905 (47.8)	1,500,815 (60.0)	1,834,203 (63.5)	195,944 (56.4)	<0.001
Smoking						<0.001
Never	267, 771 (70.1)	2,531,277 (63.8)	1,426,354 (57.0)	1,561,868 (54.1)	197,560 (56.9)	
Ex-smoker	26, 713 (7.0)	448,766 (11.3)	414,902 (16.6)	518,776 (18.0)	47,159 (13.6)	
Current	87,554 (22.9)	989,758 (24.9)	660,103 (26.4)	806,549 (27.9)	102,793 (29.5)	
Heavy drinker	17,289 (4.5)	251,275 (6.3)	206,509 (8.3)	289,162 (10.0)	36,571 (10.5)	<0.001
Regular physical activity	37,734 (9.9)	669,580 (16.9)	513,312 (20.5)	586,079 (20.3)	61,427 (17.7)	<0.001
Income in lowest 20%	82,639 (21.6)	846,583 (21.3)	503,796 (20.1)	574,288 (19.9)	74,898 (21.6)	<0.001
Diabetes mellitus	13,360 (3.5)	224,958 (5.7)	227,126 (9.1)	357,415 (12.4)	60,714 (17.5)	<0.001
Hypertension	39,684 (10.4)	694,329 (17.5)	708,653 (28.3)	1,109,621 (38.4)	178,025 (51.2)	<0.001
Dyslipidemia	21,055 (5.5)	475,335 (12.0)	494,999 (19.8)	737,213 (25.5)	107,665 (31.0)	<0.001

*Data are presented as number (percentage) or mean ± standard deviation*.

### BMI Alters the Risk of Incident TB

During the 7.3-year follow-up duration, the incidence of TB was 0.92 per 1,000 person-years in the normal weight without DM, 2.26 in the normal weight with DM, 1.80 in the underweight without DM, and 5.35 in the underweight with DM.

In participants with DM, compared with the normal weight, the underweight showed an increased risk of incident TB [adjusted hazard ratio (aHR), 2.13; 95% confidence interval (CI), 1.93–2.35]; however, overweight (aHR, 0.58; 95% CI, 0.55–0.61), obese (aHR, 0.44; 95% CI, 0.41–0.46), and severely obese (aHR, 0.29; 95% CI, 0.25–0.33) participants showed decreased risks of incident TB.

A similar trend was also observed in participants without DM. Compared with the normal weight, the underweight showed an increased risk of incident TB (aHR, 2.21; 95% CI, 2.14–2.28); however, overweight (aHR, 0.54; 95% CI, 0.53–0.55), obese (aHR, 0.36; 95% CI, 0.35–0.37), and severely obese (aHR, 0.28; 95% CI, 0.26–0.31) participants showed decreased risks of incident TB (within group HR column in Table 2).

**Table 2:** Hazard ratios and 95% confidence intervals for incident tuberculosis by diabetes mellitus and body mass index.

**Diabetes mellitus**	**Body mass index (kg/m^**2**^)**	**Number of subjects**	**Number of tuberculosis**	**Duration (PY)**	**IR (/1,000 PY)**	Within-group HR (95% CI)^*^	Overall HR (95% CI)^*^
No	<18.5	368,678	4,725	2,632,033	1.80	2.21 (2.14–2.28)	2.21 (2.14–2.28)
	18.5–22.9	3,744,843	24,902	27,196,588	0.92	1 (Reference)	1 (Reference)
	23.0–24.9	2,274,233	9,169	16,592,920	0.55	0.54 (0.53–0.55)	0.54 (0.52–0.55)
	25.0–29.9	2,529,778	7,004	18,469,674	0.38	0.36 (0.35–0.37)	0.37 (0.36–0.38)
	≥30	286,798	558	2,091,306	0.27	0.28 (0.26–0.31)	0.29 (0.27–0.31)
Yes	<18.5	13,360	448	83,771	5.35	2.13 (1.93–2.35)	3.24 (2.95–3.56)
	18.5–22.9	224,958	3,532	1,566,537	2.26	1 (Reference)	1.51 (1.46–1.57)
	23.0–24.9	227,126	2,040	1,619,120	1.26	0.58 (0.55–0.61)	0.87 (0.83–0.91)
	25.0–29.9	357,415	2,308	2,569,395	0.90	0.44 (0.41–0.46)	0.65 (0.62–0.68)
	≥30	60,714	218	438,279	0.50	0.29 (0.25–0.33)	0.42 (0.36–0.48)

* *Adjusted for age, sex, smoking status, alcohol consumption (heavy drinker or not), regular physical activity, income (lowest 20% or not), hypertension, and dyslipidemia*.

*PY, person-years; IR, incidence rate; HR, hazard ratio; CI, confidence interval*.

### Joint Effects of Underweight and DM on the Risk of Incident TB

**Figure 2:**
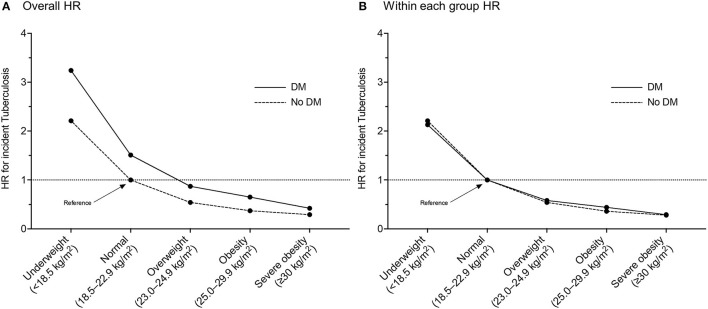
Multivariable hazard ratios of incident tuberculosis in participants with or without diabetes mellitus according to body mass index category: **(A)** with a reference of normal weight participants without diabetes mellitus; **(B)** with a reference of normal weight participants within each group by diabetes mellitus. HR, hazard ratio; DM, diabetes mellitus.

Table 2 (overall HR column) and Figure 2A reveal the joint effects of underweight and DM on the risk of incident TB. In the entire study population, compared with the normal weight without DM, gradually increased risks of incident TB were observed in the normal weight with DM (aHR, 1.51; 95% CI, 1.46–1.57), the underweight without DM (aHR, 2.21; 95% CI, 2.14–2.28), and the underweight with DM (aHR, 3.24; 95% CI, 2.95–3.56). The aHR for the joint effect of double exposure (underweight plus DM) was 3.24, similar to the expected risk assuming the independent effects of underweight and DM (1.51 ^*^ 2.21 = 3.34), which denoted no significant interaction between underweight and DM. When examined within each group by DM (DM and non-DM groups), the risk estimates by BMI status were similar in both groups (Figure 2B). Among participants with DM, DM control status (fasting blood glucose of <130 vs. ≥130 mg/dl) did not have a significant impact on the association between the risk of TB and BMI (Supplementary Table 1).

### Stratified Analyses

Table 3 shows the joint effects of BMI and DM on the risk of incident TB, which is further stratified with sex and age groups. Compared with the normal weight without DM, the male and female underweight with DM showed 3.69-fold (95% CI, 3.31–4.11) and 2.39-fold (95% CI, 1.98–2.89) increased risks of TB, respectively. Likewise, the underweight with DM aged <65 years and those aged ≥ 65 years showed 4.99-fold (95% CI, 4.40–5.67) and 2.17-fold (95% CI, 1.89–2.49) increased risks of TB compared with the normal weight without DM aged <65 and ≥ 65 years, respectively.

**Table 3:** Hazard ratios and 95% confidence intervals for incident tuberculosis according to diabetes mellitus state and body mass index plus stratified by sex and age group.

	**Diabetes mellitus**	**BMI (kg/m^**2**^)**	**Number of subjects**	**Number of tuberculosis**	**Duration (PY)**	**IR (/1,000 PY)**	HR (95% CI)^*^
Male	No	<18.5	120,428	2,507	837,401	2.99	2.31 (2.22–2.42)
		18.5–22.9	1,758,178	14,242	12,701,787	1.12	1 (Reference)
		23.0–24.9	1,355,980	5,435	9,866,048	0.55	0.50 (0.48–0.51)
		25.0–29.9	1,611,431	3,825	11,735,025	0.33	0.30 (0.29–0.31)
		≥30	165,254	227	1,201,571	0.19	0.21 (0.18–0.24)
	Yes	<18.5	8,008	338	48,632	6.95	3.69 (3.31–4.11)
		18.5–22.9	137,727	2,574	946,122	2.72	1.63 (1.56–1.70)
		23.0–24.9	144,835	1,418	1,025,068	1.38	0.87 (0.82–0.92)
		25.0–29.9	222,772	1,524	1,592,434	0.96	0.65 (0.62–0.69)
		≥30	30,690	115	220,302	0.52	0.43 (0.36–0.51)
Female	No	<18.5	248,250	2,218	1,794,632	1.24	2.09 (2.00–2.19)
		18.5–22.9	1,986,665	10,660	14,494,800	0.74	1 (Reference)
		23.0–24.9	918,253	3,734	6,726,872	0.56	0.62 (0.59–0.64)
		25.0–29.9	918,347	3,179	6,734,649	0.47	0.48 (0.46–0.50)
		≥30	121,544	331	889,735	0.37	0.40 (0.35–0.44)
	Yes	<18.5	5,352	110	35,139	3.13	2.39 (1.98–2.89)
		18.5–22.9	87,231	958	620,415	1.54	1.26 (1.18–1.35)
		23.0–24.9	82,291	622	594,052	1.05	0.85 (0.78–0.92)
		25.0–29.9	134,643	784	976,960	0.80	0.65 (0.60–0.70)
		≥30	30,024	103	217,977	0.47	0.41 (0.34–0.50)
Age <65 years	No	<18.5	323,437	3,168	2,346,776	1.35	2.26 (2.18–2.35)
		18.5–22.9	3,351,805	17,536	24,469,038	0.72	1 (Reference)
		23.0–24.9	2,000,993	6,081	14,641,246	0.42	0.52 (0.50–0.54)
		25.0–29.9	2,216,788	4,499	16,214,314	0.28	0.34 (0.33–0.35)
		≥30	258,151	383	1,884,277	0.20	0.27 (0.24–0.30)
	Yes	<18.5	7,427	241	50,412	4.78	4.99 (4.40–5.67)
		18.5–22.9	147,458	1,883	1,053,948	1.79	1.84 (1.75–1.93)
		23.0–24.9	155,710	1,107	1,126,025	0.98	1.00 (0.94–1.07)
		25.0–29.9	256,906	1,293	1,863,801	0.69	0.72 (0.68–0.77)
		≥30	47,337	139	343,752	0.40	0.47 (0.39–0.55)
Age ≥ 65 years	No	<18.5	45,241	1,557	285,257	5.46	1.86 (1.76–1.96)
		18.5–22.9	393,038	7,366	2,727,550	2.70	1 (Reference)
		23.0–24.9	273,240	3,088	1,951,674	1.58	0.62 (0.60–0.65)
		25.0–29.9	312,990	2,505	2,255,360	1.11	0.45 (0.43–0.48)
		≥30	28,647	175	207,029	0.85	0.37 (0.32–0.43)
	Yes	<18.5	5,933	207	33,359	6.23	2.17 (1.89–2.49)
		18.5–22.9	77,500	1,649	512,589	3.22	1.23 (1.16–1.30)
		23.0–24.9	71,416	933	493,096	1.89	0.75 (0.70–0.80)
		25.0–29.9	100,509	1,015	705,594	1.44	0.59 (0.55–0.63)
		≥30	13,377	79	94,527	0.84	0.37 (0.30–0.46)

* *Adjusted for age, sex, smoking status, alcohol consumption (heavy drinker or not), regular physical activity, income (lowest 20% or not), hypertension, and dyslipidemia*.

*BMI, body mass index; PY, person-years; IR, incidence rate; HR, hazard ratio; CI, confidence interval*.

Table 4 shows the joint effects of BMI and DM on the risk of incident TB, which was further stratified by smoking status and alcohol consumption. Compared with the normal weight without DM, the non-current (never and ex-) and current smoker underweight with DM showed 2.92-fold (95% CI, 2.58–3.31) and 3.83-fold (95% CI, 3.31–4.43) increased risks of TB, respectively. Additionally, the underweight with DM who were non-heavy drinkers and those who were heavy drinkers showed 3.08-fold (95% CI, 2.78–3.41) and 4.38-fold (95% CI, 3.46–5.54) increased risks of TB compared with the normal weight without DM who were non-heavy drinkers and heavy drinkers, respectively.

**Table 4:** Hazard ratios and 95% confidence intervals for incident tuberculosis according to diabetes mellitus state and body mass index plus stratified by smoking status and alcohol consumption.

	**Diabetes mellitus**	**BMI (kg/m^**2**^)**	**Number of subjects**	**Number of tuberculosis**	**Duration (PY)**	**IR (/1,000 PY)**	HR (95% CI)^*^
Non-current (never and ex-) smoker	No	<18.5	285,752	3,328	2,047,072	1.63	2.28 (2.19–2.36)
		18.5–22.9	2,819,305	17,742	20,509,865	0.87	1(Ref.)
		23.0–24.9	1,672,452	6,968	12,218,821	0.57	0.57 (0.55–0.58)
		25.0–29.9	1,810,488	5,499	13,235,434	0.42	0.40 (0.38–0.41)
		≥30	198,220	454	1,447,483	0.31	0.33 (0.30–0.36)
	Yes	<18.5	8,732	259	54,820	4.72	2.92 (2.58–3.31)
		18.5–22.9	160,738	2,287	1,124,505	2.03	1.39 (1.33–1.46)
		23.0–24.9	168,804	1,436	1,205,836	1.19	0.83 (0.78–0.88)
		25.0–29.9	270,156	1,670	1,945,397	0.86	0.62 (0.58–0.65)
		≥30	46,499	171	336,025	0.51	0.42 (0.36–0.46)
Current smoker	No	<18.5	82,926	1,397	584,961	2.39	2.13 (2.01–2.26)
		18.5–22.9	925,538	7,160	6,686,723	1.07	1(Ref.)
		23.0–24.9	601,781	2,201	4,374,099	0.50	0.47 (0.45–0.49)
		25.0–29.9	719,290	1,505	5,234,240	0.29	0.28 (0.27–0.30)
		≥30	88,578	104	643,824	0.16	0.18 (0.15–0.23)
	Yes	<18.5	4,628	189	28,951	6.53	3.83 (3.31–4.43)
		18.5–22.9	64,220	1,245	442,032	2.82	1.80 (1.70–1.92)
		23.0–24.9	58,322	604	413,285	1.46	0.98 (0.90–1.07)
		25.0–29.9	87,259	638	623,998	1.02	0.75 (0.69–0.81)
		≥30	14,215	47	102,254	0.46	0.41 (0.31–0.55)
Non-heavy drinker	No	<18.5	352,631	4,359	2,520,377	1.73	2.20 (2.13–2.27)
		18.5–22.9	3,514,055	22,718	25,532,628	0.89	1(Ref.)
		23.0–24.9	2,089,539	8,360	15,249,521	0.55	0.55 (0.53–0.56)
		25.0–29.9	2,278,389	6,372	16,638,296	0.38	0.38 (0.36–0.39)
		≥30	256,286	516	1,869,414	0.28	0.30 (0.28–0.33)
	Yes	<18.5	12,118	376	76,073	4.94	3.08 (2.78–3.41)
		18.5–22.9	204,471	3,052	1,425,634	2.14	1.47 (1.42–1.53)
		23.0–24.9	205,311	1,815	1,463,844	1.24	0.87 (0.83–0.91)
		25.0–29.9	319,642	2,032	2,298,049	0.88	0.65 (0.62–0.68)
		≥30	54,655	191	394,631	0.48	0.41 (0.36–0.47)
Heavy drinker	No	<18.5	16,047	366	111,656	3.28	2.34 (2.10–2.62)
		18.5–22.9	230,788	2,184	1,663,960	1.31	1(Ref.)
		23.0–24.9	184,694	809	1,343,399	0.60	0.48 (0.44–0.52)
		25.0–29.9	251,389	632	1,831,378	0.35	0.29 (0.26–0.31)
		≥30	30,512	42	221,892	0.19	0.18 (0.14–0.25)
	Yes	<18.5	1,242	72	7,698	9.35	4.38 (3.46–5.54)
		18.5–22.9	20,487	480	140,903	3.41	1.79 (1.62–1.96)
		23.0–24.9	21,815	225	155,276	1.45	0.83 (0.72–0.96)
		25.0–29.9	37,773	276	271,346	1.02	0.64 (0.57–0.73)
		≥30	6,059	27	43,649	0.62	0.47 (0.32–0.68)

* *Adjusted for age, sex, smoking status, alcohol consumption (heavy drinker or not), regular physical activity, income (lowest 20% or not), hypertension, and dyslipidemia*.

*BMI, body mass index; PY, person-years; IR, incidence rate; HR, hazard ratio; CI, confidence interval*.

**Figure 3:**
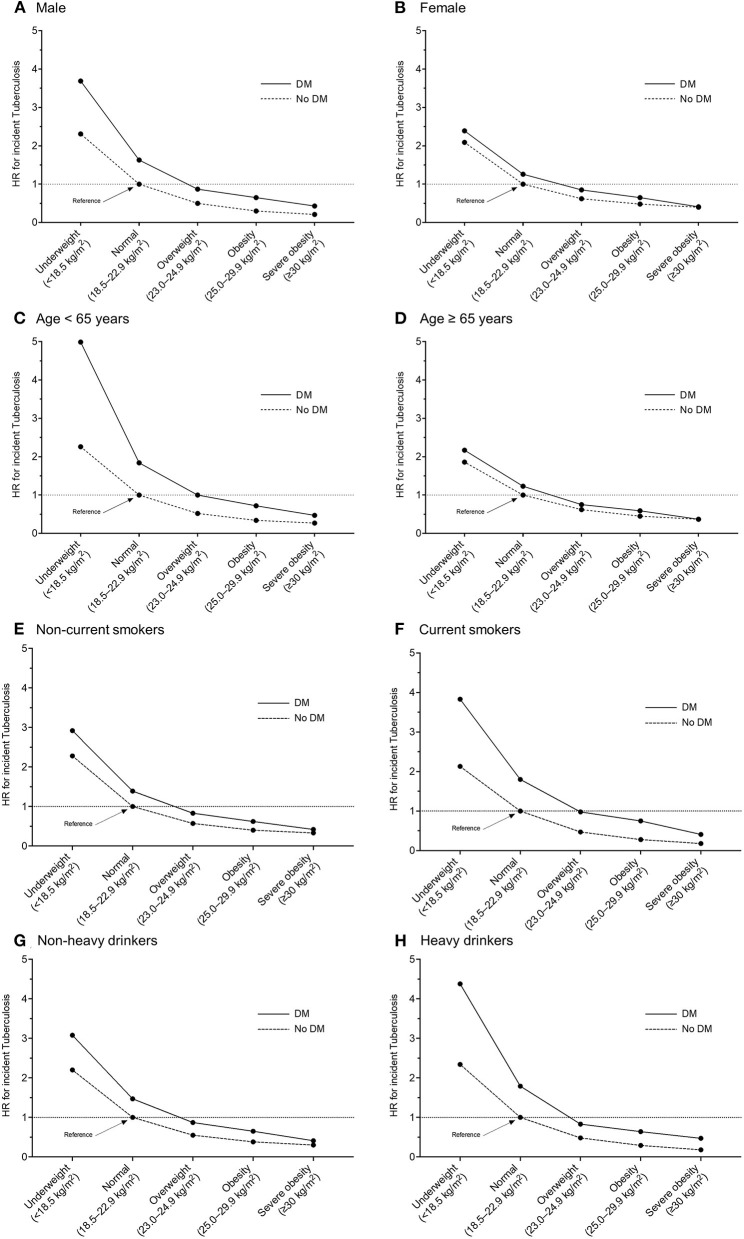
Multivariable hazard ratios of incident tuberculosis in participants with or without diabetes mellitus according to body mass index category, with a reference of normal weight participants without diabetes mellitus. Stratified by sex and age: **(A)** male, **(B)** female, **(C)** age < 65 years, **(D)** age ≥ 65 years, **(E)** non-current smokers, **(F)** current smokers, **(G)** non-heavy drinkers, and **(H)** heavy drinkers. HR, hazard ratio; DM, diabetes mellitus.

Regarding the joint effects of underweight and DM on the risk of incident TB, a multiplicative effect was observed in participants aged < 65 years, current smokers, and heavy drinkers; and there were no significant interactions between BMI and DM in males, females, those aged ≥ 65 years, non-current (never and ex-) smokers, and non-heavy drinkers (Figure 3).

## Discussion

This population-based longitudinal study evaluated the effects of DM and BMI on the risk of incident TB. Compared to the normal weight without DM, the normal weight with DM, the underweight without DM, and the underweight with DM showed a 1.5-fold, a 2.2-fold, and a 3.2-fold increased risk of TB, respectively. Although there was no significant interaction between BMI and DM on the risk of TB in the overall population, the synergistic effect of underweight and DM was evident in subgroups of aged <65 years, current smokers, and heavy drinkers.

DM is a well-known risk factor of TB development; a previous meta-analysis showed a three-fold increased risk of TB incidence (8). There have been several studies suggesting biological plausibility to explain the underlying mechanism. Studies in animal models revealed that diabetic mice had higher mycobacterial loads in the early course of *Mycobacterium tuberculosis* infection than euglycemic mice did (19, 20). Additionally, diabetic mice revealed decreased levels of interferon-γ and interleukin-12 during *M. tuberculosis* infection than euglycemic mice, which denotes diminished T helper 1 adaptive immunity (19). Furthermore, DM patients' neutrophils showed reduced chemotaxis and oxidative killing potential, indicating reduced innate immunity (21). In agreement with previous reports, our study confirms the increased risk of TB incidence in patients with DM. Additionally, DM control status did not have a significant impact on the association between TB risk in participants with DM in this study; therefore, DM itself is more important for the risk of TB compared with DM control status.

In addition to DM, underweight has also been known to increase TB incidence. A previous meta-analysis demonstrated a log-linear inverse relationship between TB incidence and BMI (4); our study also showed that underweight subjects had an increased risk of TB incidence and that the chance was inversely correlated with BMI. The possible mechanistic link between underweight and TB incidence can be explained by the fact that low nutritional status has harmful effects on a cell-mediated immune system. Experimental animal studies revealed that protein deficiency, in particular, could have devastating consequences regarding T-cell trafficking and antigen-induced proliferation, inability to form mature granulomas, and diminished production of protective cytokines (4, 22). In contrast, in line with previous reports (4, 23, 24), this study also showed that overweight and obese individuals had a significantly lower risk of TB than normal-weight individuals. Although the biological mechanism of the phenomenon is not established, leptin, an adipose tissue-driven energy-regulating hormone, might affect the T-helper 1/T-helper 2 balance of the host immune system, subsequently altering the risk of TB infection (5, 25, 26). Specifically, a considerable proportion of Asian patients with DM was underweight or normal weight, unlike Western populations, where most DM cases are attributable to obesity or overweight (10, 27). Hence, considering BMI is essential when assessing TB risk in Asian patients with DM; however, as a previous study suggested, there is a complex interplay between body weight and DM on the risk of TB (5).

In this context, we should assess the joint effects of DM and BMI on the TB risk. A recent study from Singapore also investigated a joint effect of DM and underweight on TB development compared to obesity. However, the study had two major limitations: (1) subjects with obesity, not those with normal weight, were the reference group for the analysis, and (2) the overall study population was small, which limited statistical power and further stratified analyses (10). While overcoming the limitations of the previous study, we reassessed the joint effect in a nationwide, population-based cohort. This study showed that there was no significant interaction between BMI and DM on the risk of incident TB in the overall population.

Although a stratified analysis revealed no significant joint effects of underweight and DM on TB risk in either sex group, underweight male participants showed a higher TB risk than underweight females when compared with normal weight participants in each group. In line with our findings, previous research suggested that the male predominance of TB infections was mostly attributed to socio-cultural factors leading to a higher risk of exposure to *M. tuberculosis* in males (28–30). Furthermore, this study may reinforce the support for the male predominance of TB, as a multivariable analysis was adjusted for well-known risk factors for the male predominance of TB, including alcohol abuse, smoking, and socioeconomic deprivation (31).

Interestingly, in the stratified analysis, the joint effects of underweight and DM on TB risk were synergistic only in participants younger than 65 years old. This finding may be explained by the heterogeneity of the participants without DM between the age groups. Because baseline glucose tolerance is lower in elderly participants without DM, elderly controls might have an elevated risk of incident TB compared to younger controls (32), thus reducing the evident effect of DM (8). Additionally, types of DM may affect the results. Because this study did not distinguish between type 1 and type 2 DM, the younger age group might include a higher proportion of subjects with type 1 DM, a more severe form of DM with a juvenile onset, compared to the older age group (33). Although there are some caveats when interpreting the synergistic effects of underweight and DM on TB risk, this study is meaningful in terms of providing information on whom to screen for TB. Taken together, clinicians should assess TB risk more carefully in young and thin patients with DM.

Smoking and alcohol consumption are well-known risk factors for TB (34, 35). Not surprisingly, there was also a synergistic effect between DM and BMI on the development of TB among current smokers and heavy drinkers in this study. The underweight with DM who currently smoke and drink heavily had a higher risk of TB than non-current smokers and non-heavy drinkers, respectively. Potential underlying mechanisms of high TB risk among current smokers may include decreased activity of alveolar macrophages, impaired mucociliary clearance, decreased immune response of pulmonary lymphocytes, modified pulmonary dendritic cell activity, and reduction in the cytotoxic activity of natural killer cells (36). Similarly, there are two possible pathways to explain the association between alcohol consumption and the risk of TB: impaired immune system (compromised response to newly introduced pathogens in alveolar macrophages) and aggravated malnutrition (37). Accordingly, it seems logical that smoking status and alcohol consumptions potentiate the effect of underweight on TB development in patients with DM. From a public health perspective, these findings imply that smoking cessation and avoiding heavy alcohol consumption would help improve general health and reduce the incidence of TB among underweight patients with DM.

The major strength of this study was to assess the joint effects of BMI and DM on the risk of incident TB, using a nationwide population-based longitudinal cohort with exact measurements of exposures (DM and BMI). However, there were also limitations to this study. First, the diagnoses of DM and comorbidities were based on ICD-10 codes and medications. Thus, there might be potential errors in diagnosing diseases. Second, this study was performed in Korea, an intermediate-TB-burden country. Our findings might not be generalizable to patients in other countries. Third, since we assessed the BMI of each participant upon enrollment in this study, the impact of temporal changes in BMI during the follow-up period on TB risk could not be assessed in this study. Fourth, this study could not analyze the detailed clinical courses of subjects with DM potentially affecting BMI change and TB risk, which include glucose control trajectories, types of DM, and classifications of DM medications.

In conclusion, compared to the normal weight individuals without DM, the normal weight with DM, the underweight without DM, and the underweight with DM showed a 1.5-fold, a 2.2-fold, and a 3.2-fold increased risk of TB, respectively. Although no significant interaction was observed between BMI and DM on the risk of TB among the overall population, the synergistic effect of underweight and DM was evident in participants younger than 65 years, current smokers, and heavy drinkers, suggesting that a focused screening of incident TB in patients with DM may be beneficial according to this study's results.

## Data Availability Statement

The datasets presented in this article are not readily available because restrictions apply to the availability of these data, which were used under license for this study. Requests to access the datasets should be directed to Dong Wook Shin, dwshin.md@gmail.com.

## Ethics Statement

The studies involving human participants were reviewed and approved by Institutional Review Board of Hallym University Kangnam Sacred Heart Hospital. Written informed consent for participation was not required for this study in accordance with the national legislation and the institutional requirements.

## Author Contributions

DS is the guarantor of this study. HC, JY, HL, and DS drafted the manuscript. All authors listed have provided substantial contributions to the conception, design, data acquisition, analysis, or interpretation of this work, and participated in revising the manuscript after a critical review.

## Funding

This research was supported by Basic Science Research Program through the National Research Foundation of Korea (NRF) funded by the Ministry of Science, Information, and Communications Technologies (Grant Nos. 2020R1F1A1070468 and 2021M3E5D1A01015176 to HL and 2019R1G1A1008692 to HC) and the Korean Ministry of Education (Grant No. 2021R1I1A3052416 to HC). The funders had no role in the design of the study, the collection and analysis of the data, or the preparation of the manuscript.

## Conflict of Interest

The authors declare that the research was conducted in the absence of any commercial or financial relationships that could be construed as a potential conflict of interest.

## Publisher's Note

All claims expressed in this article are solely those of the authors and do not necessarily represent those of their affiliated organizations, or those of the publisher, the editors and the reviewers. Any product that may be evaluated in this article, or claim that may be made by its manufacturer, is not guaranteed or endorsed by the publisher.
