# Supplementary material for: Body Mass Index, Diabetes, and Risk of Tuberculosis: A Retrospective Cohort Study
Source: Front Nutr. 2021 Dec 1;8:739766. doi: 10.3389/fnut.2021.739766 (PMC8671831; doi:10.3389/fnut.2021.739766)
Supplement: Supplementary file 1 [file Table_1.PDF]

Supplementary Table 1. Hazard ratios and 95% confidence intervals for incident tuberculosis by diabetes mellitus control status and body mass index

Diabetes mellitus control status	Body mass index (kg/m^2)	Number of subjects	Number of tuberculosis	Duration (PY)	IR (/1,000 PY)	Within-group HR (95% CI)*	Overall HR (95% CI)*
Well controlled (FBS <130 mg/dl)	<18.5	4,975	171	30,666	5.58	2.26 (1.92–2.65)	2.27 (1.94–2.66)
	18.5–22.9	89,158	1,350	617,976	2.18	1 (Reference)	1 (Reference)
	23.0–24.9	91,174	834	648,426	1.29	0.61 (0.56–0.67)	0.61 (0.56–0.66)
	25.0–29.9	142,625	938	1,024,392	0.92	0.46 (0.42–0.50)	0.46 (0.42–0.50)
	≥30	23,376	87	168,580	0.52	0.30 (0.24–0.38)	0.30 (0.24–0.37)
Poorly controlled (FBS ≥130 mg/dl)	<18.5	8,385	277	53,105	5.22	2.06 (1.81–2.33)	2.31 (2.03–2.63)
	18.5–22.9	135,800	2182	948,561	2.30	1 (Reference)	1.13 (1.06–1.21)
	23.0–24.9	135,952	1206	970,694	1.24	0.56 (0.52–0.60)	0.63 (0.58–0.68)
	25.0–29.9	214,790	1370	1,545,003	0.89	0.42 (0.39–0.45)	0.48 (0.45–0.52)
	≥30	37,338	131	269,699	0.49	0.27 (0.23–0.33)	0.32 (0.26–0.38)

*Adjusted for age, sex, smoking status, alcohol consumption (heavy drinker or not), regular physical activity, income (lowest 20% or not), hypertension, and dyslipidemia

FBS, fasting blood sugar; PY, person-years; IR, incidence rate; HR, hazard ratio; CI, confidence interval
